# Long-term exposure to bisphenol A or benzo(a)pyrene alters the fate of human mammary epithelial stem cells in response to BMP2 and BMP4, by pre-activating BMP signaling

**DOI:** 10.1038/cdd.2016.107

**Published:** 2016-10-14

**Authors:** Flora Clément, Xinyi Xu, Caterina F Donini, Alice Clément, Soleilmane Omarjee, Emmanuel Delay, Isabelle Treilleux, Béatrice Fervers, Muriel Le Romancer, Pascale A Cohen, Véronique Maguer-Satta

**Affiliations:** 1 Univ Lyon, Université Claude Bernard Lyon 1, Lyon, F-69008, France; 2 Univ Lyon, Université Claude Bernard Lyon 1, INSERM U1052, CNRS 5286, Centre Léon Bérard, Centre de Recherche en Cancérologie de Lyon, Lyon Cedex 08, F-69008, France; 3 Department of Tumor Escape Signaling, Centre Léon Bérard, Lyon, France; 4 Department of Cancer and Environnement, Centre Léon Bérard, Lyon, France; 5 Department of Cancer Cell Plasticity, Centre Léon Bérard, Lyon, France; 6 Centre Léon Bérard, Lyon, France; 7 CNRS GDR 3697 Micronit, Tours, France

## Abstract

Bone morphogenetic protein 2 (BMP2) and BMP4 are key regulators of the fate and differentiation of human mammary epithelial stem cells (SCs), as well as of their niches, and are involved in breast cancer development. We established that MCF10A immature mammary epithelial cells reliably reproduce the BMP response that we previously identified in human primary epithelial SCs. In this model, we observed that BMP2 promotes luminal progenitor commitment and expansion, whereas BMP4 prevents lineage differentiation. Environmental pollutants are known to promote cancer development, possibly by providing cells with stem-like features and by modifying their niches. Bisphenols, in particular, were shown to increase the risk of developing breast cancer. Here, we demonstrate that chronic exposure to low doses of bisphenol A (BPA) or benzo(a)pyrene (B(a)P) alone has little effect on SCs properties of MCF10A cells. Conversely, we show that this exposure affects the response of immature epithelial cells to BMP2 and BMP4. Furthermore, the modifications triggered in MCF10A cells on exposure to pollutants appeared to be predominantly mediated by altering the expression and localization of type-1 receptors and by pre-activating BMP signaling, through the phosphorylation of small mothers against decapentaplegic 1/5/8 (SMAD1/5/8). By analyzing stem and progenitor properties, we reveal that BPA prevents the maintenance of SC features prompted by BMP4, whereas promoting cell differentiation towards a myoepithelial phenotype. Inversely, B(a)P prevents BMP2-mediated luminal progenitor commitment and expansion, leading to the retention of stem-like properties. Overall, our data indicate that BPA and B(a)P distinctly alter the fate and differentiation potential of mammary epithelial SCs by modulating BMP signaling.

Breast cancers arising within lobules or ducts of the mammary epithelium can be divided into distinct groups, based on their molecular profiles.^1^ Epithelial stem cells (SCs) that generate ducts and lobules, as well as their direct progenitors and their microenvironment (niches), are believed to be privileged targets for transforming events, leading to the emergence of breast cancer. Deciphering their relative and respective roles in the etiology of the different breast cancer subtypes is crucial for understanding, preventing and treating this disease.

A growing body of evidence is accumulating implicating external chemicals in the development of breast cancer. Although epidemiological studies have so far only investigated the effects of a small number of chemicals identified as mammary carcinogens or as hormone disruptors, a clear association between breast cancer and polychlorinated biphenyls, polycyclic aromatic hydrocarbons, and organic solvents has been shown.^2, 3^ Of these, two of the most exhaustively studied chemicals are bisphenol A (BPA) and benzo(a)pyrene (B(a)P). BPA is a carbon-based synthetic compound with estrogen-mimetic properties,^4^ used to make a variety of common consumer plastics, sports equipment and compact disks. B(a)P, a polycyclic aromatic hydrocarbon, is mainly found in automobile exhaust fumes, cigarette smoke, and charbroiled food.^5^ BPA was shown to induce neoplastic transformation in human breast epithelial cells^6^ and to reduce the sensitivity of breast cancer cells to chemotherapy.^7^ Recent studies demonstrated that breast cancer SCs can be formed from MCF7 cells by B(a)P-induced mutations,^8^ and that this molecule also induces lung carcinogenesis.^5^ Hence, carcinogen-caused dysregulations to epithelial cells and/or to the cellular microenvironment could represent a driving force to promote transformation and define tumor subtype.^9, 10^

The behavior of SCs may be altered following the dysregulation of a number of signaling pathways that drive cell division, survival, commitment and differentiation.^11^ However, it is still unclear how these pathways participate in tumor initiation at the molecular level, through their regulation of the SC compartment. BMPs, members of the transforming growth factor beta (TGF*β*) superfamily, are involved in many regulatory mechanisms, including in the nervous system, prostate, skin, intestine, ovary and mammary gland. The binding of soluble BMPs to BMP receptor 2 (BMPR2) leads to the phosphorylation of the serine–threonine receptors BMPR1a and BMPR1b, which phosphorylate SMAD1/5/8 effectors.^12^ This subsequently results in the formation of a complex with the SMAD4 cofactor that translocates to the nucleus to regulate gene expression.^13, 14^

In cancer, the function of BMPs remains controversial, as it was shown both to stimulate and inhibit tumor cell growth.^15^ Soluble BMPs and alterations of several components of the BMP signaling pathways have been detected in breast cancer and were shown to impact the behavior of cancer cells.^16, 17, 18^ Although current data mainly implicate BMPs in late stages of tumorigenesis and metastasis, we demonstrated both *in vitro* and *in vivo* that chronic exposure of immature epithelial cells to BMP2 promotes their malignant transformation in an inflammatory context, at a very early stage.^9^ Our data suggested that high levels of BMP2 in the luminal tumor microenvironment could be produced by mammary fibroblasts in response to exposure to environmental pollutants, such as radiation or estrogen-mimetic molecules (BPA), which were able to shift the balance of secreted BMP molecules in favor of BMP2.^9^ These events, affecting both the niche and their resident epithelial cells, create optimal conditions for the promotion of malignant transformation and progression by BMP2.^19^ However, the effects of pollutants on BMP signaling in mammary epithelial cells have not yet been investigated. Here, we examined whether BPA or B(a)P could directly alter immature mammary epithelial cell features and their response to BMPs. Our data indicate that BPA or B(a)P by themselves do not significantly alter the properties of epithelial SCs. However, they modify the response of cells to BMPs soluble molecules by changing their sensitivity to BMP signaling, by modulating type-1 receptors localization and downstream signal priming, and by altering the fate and differentiation of SCs in response to BMP2 or BMP4.

## Results

### MCF10A cells reliably reproduce the response of human immature mammary primary epithelial cells to BMP2 and BMP4

**Figure 1:**
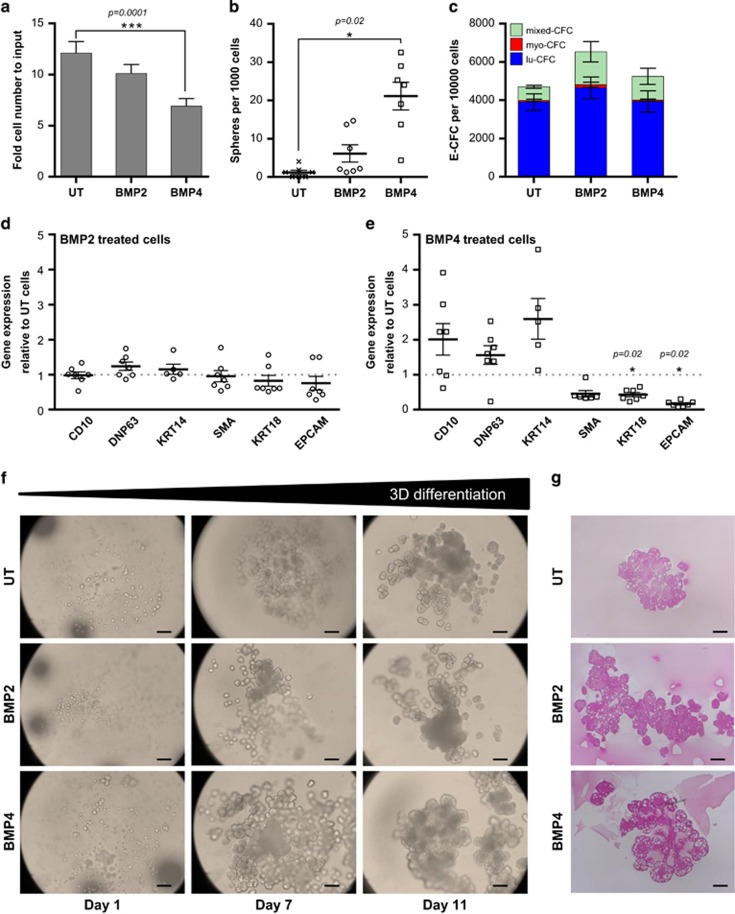
MCF10A is a reliable model of human immature cells regarding their response to BMP2 and BMP4. (**a**) Bar graph showing cell proliferation of MFC10A cells treated for 4 days with BMP2 or BMP4 (15 ng/ml). UT are the untreated cells. Data are represented as mean +/− S.E.M., *n*=21. *P=0.0001 indicates difference between UT and BMP4 treated cells. (**b**) Column scatter plot showing the number of spheres obtained after 1 week for 1000 seeded cells. One hundred single MFC10A cells were seeded in 96 ultra-low attachment plates (150 *μ*l medium/well) after 4 days of treatment with BMP2 or BMP4 (15 ng/ml), each point represents the mean of one experiment (mean of 30 wells). UT are the untreated cells. Data are represented as mean +/− S.E.M., *n*=7. ***P=0.02 indicates difference between UT and BMP4 treated cells. (**c**) After 4 days of BMP2 or BMP4 (15 ng/ml) treatment, 200 cells per well were seeded in 12-well plates in triplicate in E-CFC assays for 5–6 days. Bar graph showing the number of different types of E-CFC obtained per 10 000 seeded cells (mixed colonies, mixed-CFC; myoepithelial colonies, myo-CFC; luminal colonies, lu-CFC). UT are the untreated cells. Data are represented as mean +/− S.E.M., *n*=6. (**d**,**e**) Cells were treated with BMP2 (**d**) or BMP4 (**e**) (15 ng/ml) for 4 days, before conducting quantitative PCR (qPCR) analyses to detect the level of expression of differentiation markers. Column scatter plot showing arbitrary units of the ratio to untreated (UT) cells. Data represent mean +/− S.E.M., *n*=5. (**f**) Bright-field images at day 1, 7 or 11 of 3D structures generated in the TDLU assay from untreated, or BMP2 or BMP4 previously treated (4 days, (15 ng/ml)) cells. Data are representative of *n*=9 independent experiments, scale bars=100 *μ*m. (**g**) Immunohistochemistry of representative TDLU sections, stained for hematoxylin and eosin. Data are representative of *n*=9 independent experiments, scale bars=100 *μ*m

We initially evaluated whether the response of MCF10A cells^20^ to BMP2 and BMP4 treatment was representative of the behavior of human primary mammary progenitors/SCs, as reported.^9^ After confirming the similarity in the gene expression profiles of MCF10A cells and primary unsorted cells derived from normal mammoplasties (Suppplementary Figure 1a), we assessed the viability, proliferation and ability of MCF10A cells to generate spheres, colonies, and terminal duct lobular units (TDLU) after 4 days of treatment with BMP2 or BMP4 (15 ng/ml). Although cell viability was high, irrespective of the treatment administered, indicating that neither BMP2 nor BMP4 were immediately toxic to cells (Supplementary Figure 1b), BMP4 significantly decreased cell proliferation (Figure 1a). A similar trend was observed in the case of BMP2, though this was not statistically significant. These results corroborate data obtained with primary human mammary epithelial cells (Supplementary Figure 1c). As cell viability was not affected, we evaluated whether BMP2 and BMP4 inhibited cell proliferation by inducing cell differentiation. To do so, we investigated the impact of BMPs on the ability of MCF10A cells to generate spheres or epithelial progenitors. Indeed, stem-like cells are known to have the unique properties of growing under low adherence conditions and of forming spheres when seeded in limiting dilutions, properties lost in differentiated cells.^21^ We observed that BMP4 significantly increased the ability of MCF10A cells to generate spheres (20-fold) under these conditions, whereas BMP2-treated cells only experienced a fivefold increase (Figure 1b). Furthermore, results obtained from the quantification of epithelial progenitors (epithelial-colony forming cell – E-CFC assay), using a protocol adapted for human cell lines (Supplementary Figure 1d),^22^ were consistent with those previously obtained using primary cells. BMP2 treatment of MCF10A cells increased total colony number, by expanding the luminal and mixed progenitor compartments. BMP4 had no significant effect on MCF10A progenitors, despite a slight increase in mixed E-CFCs (Figure 1c). We then validated these observations by analyzing the levels of lineage markers, namely immature (cluster of differentiation 10 – CD10, deltaNp63 – DNP63), myoepithelial (keratin 14 – KRT14, smooth muscle actin – SMA) and luminal (KRT18, epithelial cell adhesion molecule – EPCAM), following BMP2 or BMP4 treatment of MCF10A cells. Although BMP2 had no significant effect (Figure 1d), BMP4-treated cells displayed a higher level of immature markers (CD10, DNP63) and a lower level of more differentiated markers, KRT18 and EPCAM (Figure 1e). These results are consistent with findings using fresh human primary samples, confirming that BMP2 preferentially targets the luminal progenitor compartment, whereas BMP4 inhibits proliferation and differentiation of immature cells.^9^

To confirm that BMP4 preserves stem-like properties of treated cells, we conducted TDLU assays, which evaluate the ability of mammary SCs to form ducts and lobular structures in a 3 dimensional (3D) substrate. Kinetic analyses of these assays and the subsequent paraffin embedding, sectioning and H&E staining of structures formed, revealed that BMP2 had little effect on 3D structure appearance (Figure 1f) but increased the number of lobules formed compared with untreated MCF10A cells (Figure 1g). Conversely, BMP4 accelerated the formation of TDLU structures (Figure 1f) and led to the generation of well-organized structures with complex ducts and lobules (Figure 1g). Overall, these data confirm that BMP2 and BMP4, despite their structural homology, distinctly modulate the fate of human immature mammary epithelial cells^9^ and demonstrate that the MCF10A cell line constitutes an adequate model to study regulatory mechanisms underlying the response of these cells to BMPs.

### Impact of BPA and B(a)P on immature mammary epithelial cells

**Figure 2:**
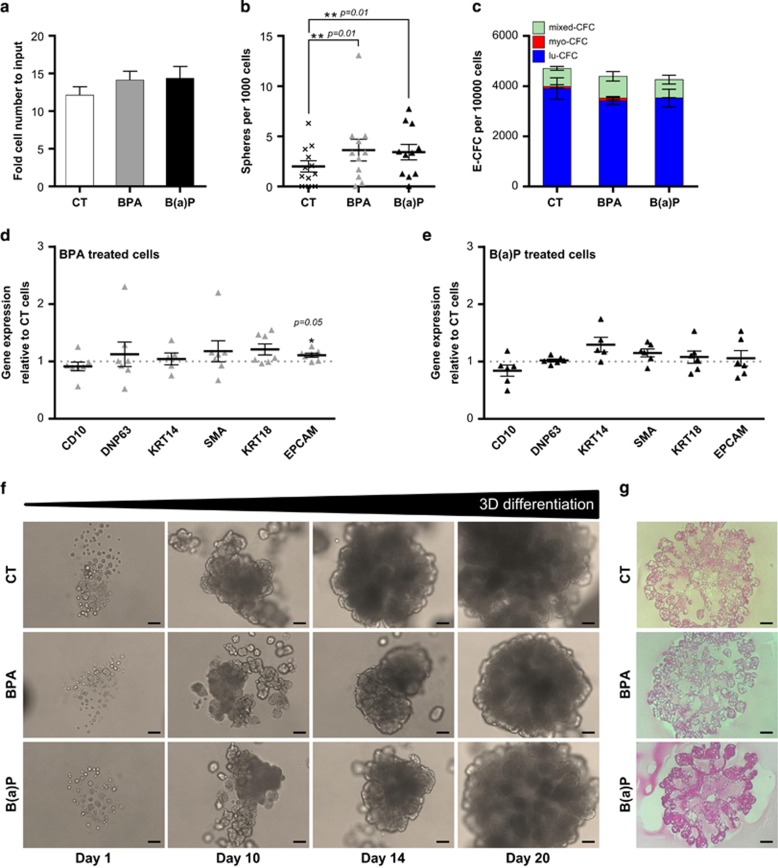
Pollutants alone do not affect MCF10A immature features. (**a**) Bar graph showing cell proliferation of MFC10A cells exposed 60 days to BPA or B(a)P (10^−10^ M). CT are the control cells (unexposed to pollutants). Data are represented as mean +/− S.E.M., *n*=21. (**b**) Column scatter plot showing the number of spheres obtained after 1 week for 1000 seeded cells. One hundred single MFC10A cells exposed 60 days to BPA or B(a)P (10^−10^ M) were seeded onto 96 ultra-low attachment plates (150 *μ*l medium/well), each value represents the mean of one experiment (mean of 30 wells). CT are the control cells (unexposed to pollutants). Data are represented as mean +/− S.E.M., *n*=11. **P=0.01 indicates difference between CT and treated cells. (**c**) After 60 days of exposure to BPA or B(a)P (10^−10^ M), 200 cells per well were seeded onto 12-well plates in triplicate to perform E-CFC assays for 5–6 days. Bar graph showing the number of different types of E-CFCs obtained per 10 000 seeded cells (mixed colonies, mixed-CFC; myoepithelial colonies, myo-CFC; luminal colonies, lu-CFC). CT are the control cells (unexposed to pollutants). Data are represented as mean +/− S.E.M., *n*=6. (**d**,**e**) Cells were exposed 60 days to BPA (**d**) or B(a)P (**e**) (10^−10^ M), before conducting qPCR analyses to detect the level of expression of differentiation markers. Column scatter plot showing arbitrary units of the ratio to control (CT) cells. Data represent mean +/− S.E.M., *n*=5. (**f**) Bright-field images at day 1, 10, 14 or 20 of 3D structures generated in the TDLU assay from control cells (CT), BPA or B(a)P previously exposed (60 days, (10^−10^ M)) cells. Data are representative of *n*=15 independent experiments, scale bars=100 *μ*m. (**g**) Immunohistochemistry of representative TDLU sections, stained for hematoxylin and eosin. Data are representative of *n*=15 independent experiments, scale bars=100 *μ*m

We previously showed that bisphenols are likely involved in the emergence of the luminal breast cancer by inducing an abnormal synthesis of BMP2 by fibroblasts and stromal cells.^9, 23^ Here, we investigated whether chronic exposure to BPA or B(a)P commonly found in our environment, affected the behavior of immature mammary epithelial cells. We treated MCF10A cells for 60 days with BPA or B(a)P (both at a final concentration of 10^−10^ M), leading to the establishment of three distinct cell sublines. We observed a slight increase in cell proliferation on exposure to pollutants (Figure 2a) and no effects on cell viability (data not shown). Cells chronically exposed to BPA or B(a)P displayed a modest but significant increase in their sphere-forming capacity compared with MCF10A control cells (Figure 2b). We did not detect any differences in the E-CFC output between MCF10A control cells and cells exposed to BPA or B(a)P (Figure 2c). Moreover, we observed very little impact of pollutant exposure at the transcriptional level (Figures 2d and e), except a slight increase in the expression of mature lineage markers (SMA, KRT18 and EPCAM) on BPA exposure (Figure 2d). In contrast to BPA, B(a)P only modestly increased the expression of myoepithelial markers (KRT14 and SMA) (Figure 2e). Lastly, we observed no differences in the kinetic appearance or structures of TDLU in cells exposed to pollutants compared with control cells (Figures 2f and g for histological confirmation). Therefore, our data indicate that long-term exposure to either BPA or B(a)P does not significantly modify the properties of immature cells.

### BPA and B(a)P modify the expression and localization of type-1 BMP receptors in MCF10A cells

**Figure 3:**
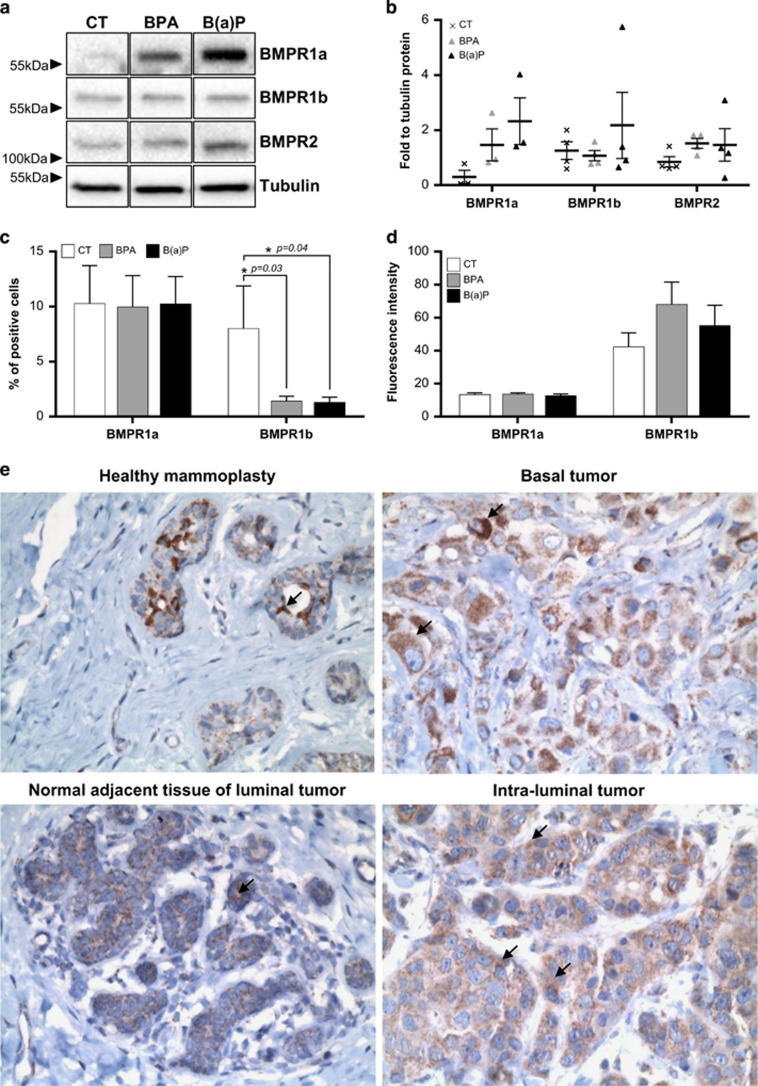
BPA and B(a)P modify the level of expression and localization of BMP receptors. (**a**) Western blot bright-field images of MCF10A cells exposed 60 days to BPA or B(a)P (10^-10^ M) CT are the control cells (unexposed to pollutants). Data represent four independent experiments. (**b**) Column scatter plot showing the quantification of proteins of interest detected by western blot normalized against the level of tubulin. Data are represented as mean +/− S.E.M., *n*=4. (**c**,**d**) Bar graph showing the percentage (**c**) or mean fluorescence intensity (**d**) of BMP receptors 1a- or 1b-positive MCF10A cells exposed 60 days to BPA or B(a)P (10^−10^ M), by flow cytometry. Data are represented as mean +/− S.E.M., *n*=11. (**e**) Immunohistochemistry of BMPR1b receptor in a healthy mammoplasty, normal adjacent tissue of luminal tumor, basal or luminal tumor slides, scale bars=20 *μ*M

We previously showed that BMP receptors, and especially type 1b receptor, mediate the BMP-driven response during SCs regulation, transformation and maintenance in different tissues.^9, 24, 25^ Here, we analyzed the impact of pollutants on BMP receptors in MFC10A cells. On long-term exposure to BPA or B(a)P, protein levels detected by western blot revealed that although BMPR1b remained unaffected, an increase in BMPR1a (two-fold) and BMPR2 (only slightly) levels could be observed (Figures 3a and b). Surprisingly, BMPR1a levels at the cell surface remained unchanged, as indicated both by the percentage of positive cells (Figure 3c) and by the mean fluorescence intensity obtained by flow cytometry (Figure 3d). Conversely, the percentage of cells expressing BMPR1b at their membrane strongly and significantly decreased on exposure to BPA and B(a)P (Figure 3c). However, although fewer cells expressed BMPR1b at their surface, the ones that retained their expression presented more molecules of BMPR1b per cell, as indicated by an increase in the mean fluorescence intensity (Figure 3d). These findings suggest that BPA and B(a)P modify the expression and localization of BMP type-1 receptors, which is likely to impact BMP-mediated biological functions. Interestingly, although the BMPR1b receptor is primarily localized in the apical membrane of luminal cells in healthy mammoplasties, as well as in the normal adjacent tissue of luminal tumors, we observed by immunohistochemical staining a different cellular localization of BMPR1b in tumor tissues (Figure 3e). Indeed, BMPR1b was mostly detected in the cytoplasm of both basal and luminal tumor cells and its localization was more diffuse than in healthy tissues (Figure 3e). Hence, exposure to pollutants increased levels of type-1 receptors detected by western blot and decreased those obtained by flow cytometry, suggesting that BPA and B(a)P could also modify the localization of these receptors, initiating dysregulations similar to those observed in cancer tissues.

### BPA and B(a)P pre-activate the MFC10A response to BMP2 and BMP4 by changing the expression and distribution of BMP receptors

**Figure 4:**
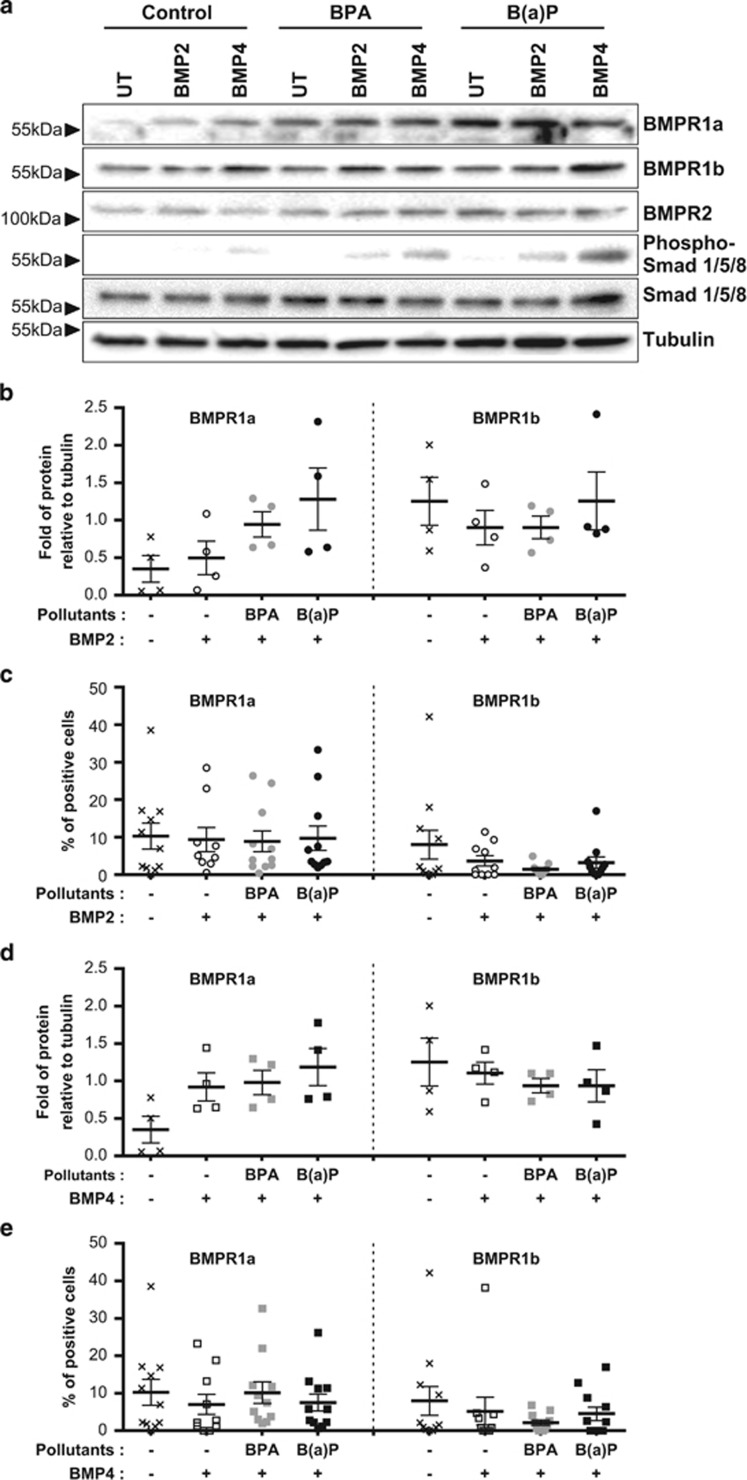
Signal transduction in response to BMP2 and BMP4 is affected by long-term exposure to BPA and B(a)P. (**a**) Western blot bright-field images of MCF10A cells exposed 60 days to BPA or B(a)P (10^−10^ M) and treated for 4 days with BMP2 or BMP4 (15 ng/ml). UT are the BMP-untreated cells. Data are representative of four independents experiments. (**b**) Column scatter plot showing the quantification of BMP receptors 1a (left panel) or 1b (right panel) detected by western blot normalized against the level of tubulin. BPA or B(a)P-exposed cell lines were treated for 4 days with BMP2 (15 ng/ml). Data are represented as mean +/− S.E.M., *n*=4. (**c**) Column scatter plot showing the percentage of BMP receptors 1a- (left panel) or 1b-positive MCF10A cells (right panel) exposed 60 days to BPA or B(a)P (10^−10^ M) and treated for 4 days with BMP2 (15 ng/ml) by flow cytometry. Data are represented as mean +/− S.E.M., *n*=11. (**d**) Column scatter plot showing the quantification of BMP receptors 1a (left panel) or 1b (right panel) detected by western blot normalized against the level of tubulin. BPA or B(a)P-exposed cell lines were treated for 4 days with BMP4 (15 ng/ml). Data are represented as mean +/− S.E.M., *n*=4. (**e**) Column scatter plot showing the percentage of BMP receptors 1a- (left panel) or 1b-positive MCF10A cells (right panel) exposed 60 days to BPA or B(a)P (10^−10^ M) and treated for 4 days with BMP4 (15 ng/ml) by flow cytometry. Data are represented as mean +/− S.E.M., *n*=11

Next, we evaluated the BMP response of cells treated with BMP2 or BMP4 and chronically exposed to BPA and B(a)P. Although treatment of immature cells with BMP2 alone did not profoundly alter the level of BMPR1a, pre-exposure to BPA or B(a)P led to an increase in the expression of this receptor (Figures 4a and b, left panel). However, this result was not accompanied by an accumulation of this receptor at the cell surface (Figure 4c, left panel). Treatment with BMP2 alone resulted in a decrease both in the level of total protein and of BMPR1b at the cell surface (Figures 4a and b, right panel and 4c, right panel). These levels, particularly those of the total protein, were slightly affected by pre-exposing cells to B(a)P (Figures 4a–c right panel). Following exposure to BPA, the level of total protein remained unaltered (Figures 4a and b, right panel), whereas the cell-surface expression dropped (Figure 4c, right panel). These findings indicate that the combined exposure to treatment with pollutants and BMP2 favors a cytoplasmic expression of BMPR1a and BMPR1b. Moreover, we observed a lack of correlation between the total amount of receptors detected by western blot (Figures 4a and d) and by flow cytometry (Figure 4e) in BMP4-treated cells. The amount of BMPR1a increased following long-term exposure to B(a)P (Figure 4d, left panel), although this was not observed at the cell membrane level (Figure 4e, left panel). Finally, the level of BMPR1b protein initially decreased on BMP4 treatment, and exposure to both pollutants revealed no major changes (Figures 4d and e, right panel), except for the decrease observed on exposure to BPA using both techniques. These data suggest that BPA may favor a BMPR1a-mediated BMP4 response.

**Figure 5:**
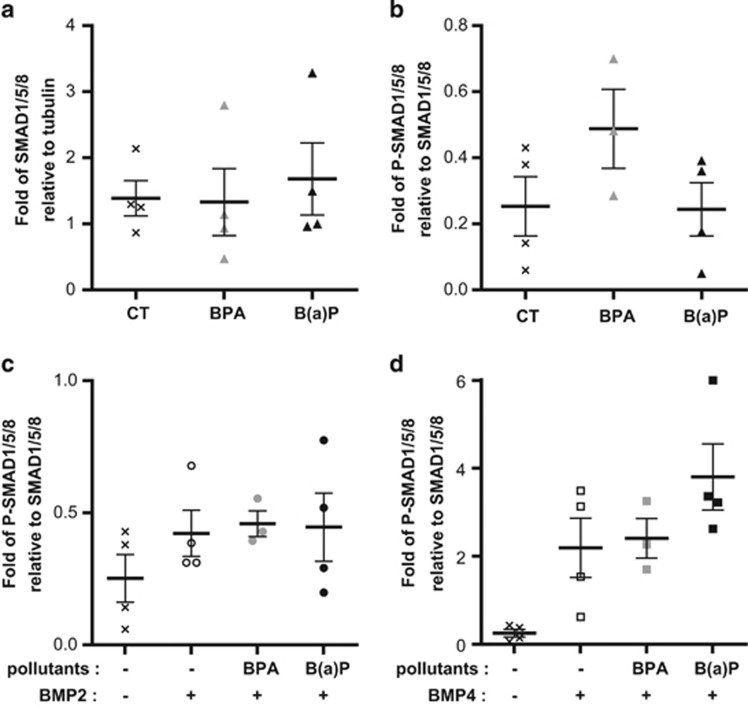
SMAD1/5/8 is pre-activated by BPA and response to BMP4 is amplified following long-term exposure to BPA and B(a)P. (**a**) Column scatter plot showing the quantification of SMAD1/5/8 detected by western blot normalized against the level of tubulin, in MCF10A cells exposed 60 days to BPA or B(a)P (10^−10^ M). CT are the control cells (unexposed to pollutants). Data are represented as mean +/− S.E.M., *n*=4. (**b**) Column scatter plot showing the quantification of Phospho-SMAD1/5/8 proteins normalized against SMAD1/5/8 proteins detected by western blot in MCF10A cells exposed 60 days to BPA or B(a)P (10^−10^ M). Data are represented as mean +/− S.E.M., *n*=3. (**c**) Column scatter plot showing the quantification of phospho-SMAD1/5/8 proteins normalized against SMAD1/5/8 proteins detected by western blot in MCF10A cells exposed 60 days to BPA or B(a)P (10^−10^ M) and treated for 4 days with BMP2 (15 ng/ml). Data are represented as mean +/− S.E.M., *n*=4. (**d**) Column scatter plot showing the quantification of phospho-SMAD1/5/8 proteins normalized against SMAD1/5/8 proteins detected by western blot MCF10A cells exposed 60 days to BPA or B(a)P (10^−10^ M) and treated for 4 days with BMP4 (15 ng/ml). Data are represented as mean +/− S.E.M., *n*=4

Exposure to B(a)P only slightly increased the total amount of SMAD1/5/8 (Figure 5a), whereas exposure to BPA increased the level of phosphorylated SMAD relative to SMAD1/5/8 (Figure 5b) without directly affecting total protein levels (Figure 5a). This suggests that, in addition to modulating the expression and localization of receptors, pollutants pre-activate the BMP signaling pathway. This is further sustained by our results that unveil a hyper activation of the SMAD phosphorylation cascade on treatment with BMP2 (Figure 5c) and especially BMP4 (Figure 5d) in cells pre-exposed to BPA or B(a)P. Altogether, our observations indicate that long-term exposure to BPA and B(a)P modifies the response of immature cells to BMP2 and BMP4 by changing the level and cellular distribution of BMP type-1 receptors, and by pre-activating SMAD1/5/8 signaling molecules through their sustained phosphorylation.

### Exposure to BPA and B(a)P primes MCF10A cells to respond to BMP2 and BMP4

**Figure 6:**
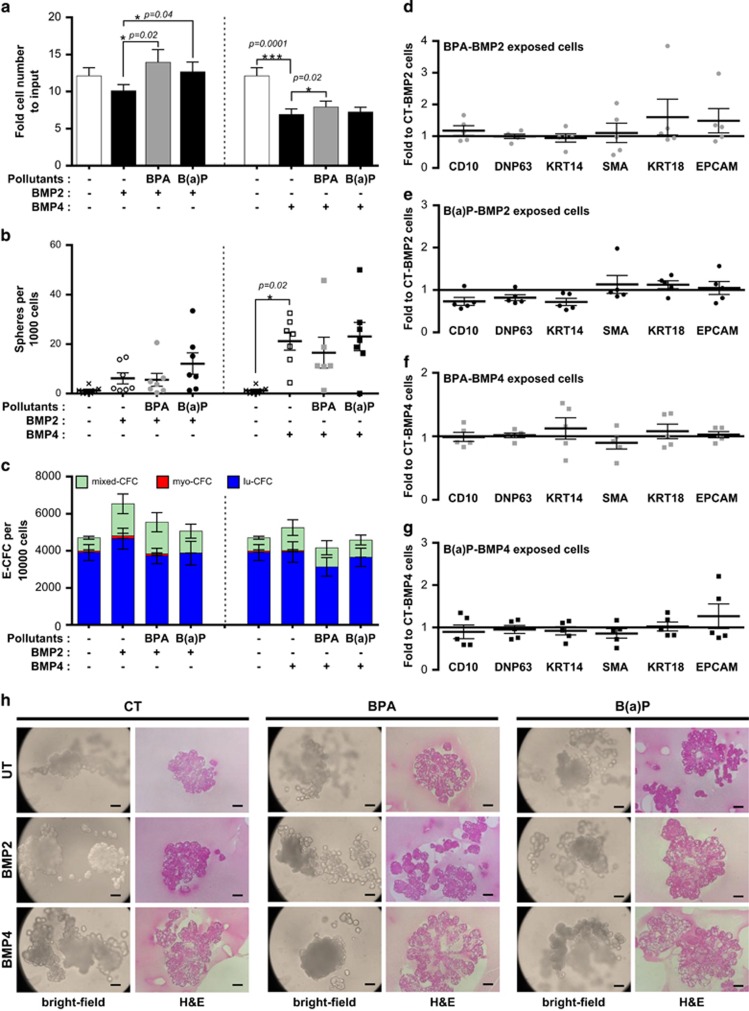
Functional effects of exposure to BPA and B(a)P on the response of immature cell to BMP2 and BMP4. (**a**) Bar graph showing cell proliferation of MFC10A cells exposed 60 days to BPA or B(a)P (10^−10^ M) and treated for 4 days with BMP2 or BMP4 (15 ng/ml). Data are represented as mean +/− S.E.M., *n*=21. (**b**) Column scatter plot showing the number of spheres obtained after 1 week per 1000 seeded cells. One hundred single MFC10A cells exposed 60 days to BPA or B(a)P (10^−10^ M) and treated for 4 days with BMP2 or BMP4 (15 ng/ml) were seeded onto 96 ultra-low attachment plates (150 *μ*l medium/well), each value represents the mean of one experiment (mean of 30 wells). Data are represented as mean +/− S.E.M., *n*=4. (**c**) After 60 days of exposure to BPA or B(a)P (10^−10^ M) and 4 days of treatment with BMP2 or BMP4 (15 ng/ml), 200 cells per well were seeded onto 12-well plates in triplicate to perform E-CFC assays for 5–6 days. Bar graph showing the number of different types of E-CFCs obtained per 10 000 seeded cells (mixed colonies, mixed-CFC; myoepithelial colonies, myo-CFC; luminal colonies, lu-CFC). Data are represented as mean +/− S.E.M., *n*=6. (**d**,**e**) Cells were exposed 60 days to BPA (10^−10^ M) (**d**) or to B(a)P (10^−10^ M) (**e**) and treated for 4 days with BMP2 (15 ng/ml), before conducting qPCR analyses to detect the level of expression of differentiation markers. Column scatter plot showing arbitrary units of the ratio to control cells unexposed to pollutants (CT) but treated for 4 days with BMP2 (15 ng/ml). Data represent mean +/− S.E.M., *n*=5. (**f**,**g**) Cells were to BPA (10^−10^ M) (**f**) or to B(a)P (10^−10^ M) (**g**) and treated for 4 days with BMP4 (15 ng/ml), before conducting qPCR analyses to detect the level of expression of differentiation markers. Column scatter plot showing arbitrary units of the ratio to cells unexposed to pollutants (CT) treated for 4 days with BMP2 (15 ng/ml). Data represent mean +/− S.E.M., *n*=5. (**h**) Bright-field (left) or immunohistochemistry (right) images at day 10 of 3D structures generated in the TDLU assay from unexposed control cells (CT), BPA or B(a)P previously exposed (60 days, (10^−10^ M)) cells treated for 4 days with BMP2 or BMP4 (15 ng/ml). Data are representative of *n*=9 independent experiments, scale bars=100 *μ*m

Lastly, we investigated the impact of BPA and B(a)P on the response of MCF10A cells to BMP2 and BMP4 at a functional level. Despite preventing the decrease in cell proliferation following BMP2 treatment (Figure 6a, left panel), BPA and B(a)P had no effect on the BMP4-mediated decrease (Figure 6a, right panel). Pre-exposure to B(a)P amplified BMP2-mediated spheres formation (Figure 6b, left panel), whereas exposure to BPA dampened the BMP4-mediated sphere-forming ability of MCF10A cells (Figure 6b, right panel). Both BPA and B(a)P impeded the BMP2-mediated luminal progenitor expansion (Figure 6c, left panel), and B(a)P alone prevented the expansion of mixed colonies. However, no clear differences were observed at the gene expression level (Figures 6d–g). Surprisingly, although BPA had no effect by itself (Figure 2c), the addition of BMP4 enhanced the effect of BPA on more committed luminal and myoepithelial colonies (Figure 6c, right panel). B(a)P displayed a similar, yet weaker effect on the BMP4-mediated progenitor response (Figure 6c, right panel). This suggests that pollutants may prevent myoepithelial progenitor expansion and favor further differentiation of cells following BMP4 signaling. This is sustained by the fact that fewer spheres were formed on BPA exposure (Figure 6b, right panel) and that B(a)P prevented the BMP4-mediated increase in the expression of immature genes (CD10, DNP63) (Figure 6g).

Finally, the impact of pollutants on BMPs control of SCs pluripotent differentiation was analyzed using the TDLU assay. Results further confirmed that BPA preferentially affects BMP4-controlled SCs features, such as sphere-forming ability, resulting in fewer TDLU structures (Figure 6h, center panel). Conversely, BPA did not reproducibly impair BMP2-mediated lobule structures, whereas exposure to B(a)P markedly prevented this phenomenon (Figure 6h, right panel). In this case, B(a)P-exposed cells displayed enhanced immature properties in the presence of BMP2, such as the ability to from highly organized ducts and lobule structures compared with BMP2-treated MCF10A cells, which mainly formed lobules (Figure 6h, left panel). This is consistent with the greater amount of spheres and the fewer E-CFC progenitors obtained (Figures 6b and c). Altogether, our data revealed that pollutants modulate the response of MCF10A cells to BMP2 and BMP4 treatment. Indeed, BPA prevented SC maintenance by BMP4 and promoted cell differentiation towards a myoepithelial phenotype, whereas B(a)P prevented the BMP2-mediated luminal progenitor commitment and expansion, thus enhancing the retention of SC features.

## Discussion

Pollutants, particularly BPA, were shown to promote proliferation, apoptosis and transformation of breast cancer cells.^3, 8, 10, 26, 27, 28^ Although extensively studied in the context of lung cancer,^5^ the effects of B(a)P on mammary cells have barely been investigated. Previously, we showed that BPA is probably involved in facilitating the origin of luminal breast cancer by dysregulating BMP2 production by mammary fibroblasts and stromal cells.^9, 23^ Interestingly, it has recently been proposed that the physical trapping of BMP2 in extracellular matrix changes its effect on SC fate and on the differentiation of immature cells sheltered in their niche.^29^ With regards to the morphogenesis of ducts and lobules, which constitute the basic unit of the mammary gland, it was reported that changes in the stroma and its extracellular matrix lead to altered ductal morphogenesis. In addition, *in vivo* exposure to BPA during gestation and lactation increased the sensitivity to mammotropic hormones, suggesting a plausible explanation for the increased incidence of breast cancer.^30^

Here, we investigated the effects of pollutants (BPA and B(a)P) on the regulation of immature mammary cells by evaluating the impact of pollutants on the response of epithelial cells to BMP2 and BMP4. We demonstrated in MCF10A cells that BMP2 enhances the production of luminal progenitors, whereas BMP4 prevents the differentiation of this model. Indeed, we confirmed that BMP4 was more efficient at decreasing the proliferation of MCF10A cells and at re-directing these cells towards a more immature phenotype compared with BMP2. Interestingly, different assays revealed that the effects of BMP2 and BMP4 are distinct and almost exclusively modified by B(a)P and BPA, respectively. These findings corroborate data obtained using normal primary tissue of human mammary glands, showing that BMP2 and BMP4 present in the SC niche distinctly regulate mammary stem/progenitor cell fate.^9^

Many reports have highlighted the ability of BMP4 to suppress cell proliferation, whereas facilitating cell migration and relapse,^31, 32, 33^ and also have demonstrated the implication of BMPs in promoting cell migration and invasion in breast cancer patients.^34^ It was recently shown that BMP4 could also inhibit breast cancer metastasis, in particular by affecting surrounding immune cells.^35^ The effects of BMPs are thus complex to decipher, as they are context-dependent and vary according to organ or cell types, culture conditions, BMPs doses used and receptor availability. Moreover, it is important to recall that during an entire lifespan, mammary epithelial SCs can be exposed both to BMPs and/or pollutants over a prolonged period of time. Here, we analyzed the impact of long-term exposure to low doses of BPA and B(a)P, which currently represent bio-available concentrations reported to be present in food, water or environmental carriers, and their resulting impact on the BMP response. Our data revealed that pollutants could modify the physiological control of human epithelial SCs by BMPs. Indeed, we observed that BPA increased immature features and amplified the response of cells to BMPs, whereas B(a)P appeared to have a role on the progenitor compartment and to inhibit the BMP4-mediated effect on SCs. Importantly, we also uncovered that BPA and B(a)P are able to modulate the response of immature cells to BMPs, possibly by changing the expression and localization of type-1 BMP receptors. Similar mechanisms have been described in different tumor types, particularly in breast and lung cancer, for tyrosine kinase receptors, such as EGFR and IGFR1.^36, 37^ This is the first time that experimental results suggest that pollutants may be able to initiate such a modification in the localization of BMP receptors. Furthermore, BPA or B(a)P induced a hyper sensitivity of the cells to BMP ligands, as indicated by a sustained SMAD1/5/8 phosphorylation observed in response to BMP2 or BMP4 treatment. This sustained SMADs phosphorylation could modify BMP signaling, leading to different cell responses as shown for MAPK phosphorylation.^38, 39^ Therefore, this could change epithelial cell fate as reported for a long-term or serial pulse of TGF*β* stimulation.^40^ Pollutants could then alter the response of SCs to BMPs by modulating the duration of the signals.

In conclusion, BPA and B(a)P differently affect the properties of human immature mammary cells and their response to BMPs. We have provided an insight into the effects of BPA and B(a)P on the response of SCs to BMP2 and BMP4, which could contribute to very early stages of breast cancer initiation. Indeed, it is believed that exposure to pollutants exacerbates the pro-tumoral effects of BMPs. Thus, monitoring pollutant exposure and BMP pathway activation may be helpful to assess the risk of tumor initiation especially in breast tissue.^41^ As people are chronically exposed to low concentrations of various pollutants, including BPA and B(a)P, our data also highlight that the combined targeting of pollutants and BMPs effects may be a new avenue to develop novel preventive and therapeutic strategies.

## Materials and Methods

### Cell lines and functional assays

MCF10A cells were purchased from the ATCC and cultured according to the manufacturer's recommendations in phenol red-free Dulbecco's modified Eagle's medium (DMEM)/F-12 nutrient mix supplemented with 5% horse serum (Life Technologies, France), 10 *μ*g/ml insulin, 0.5* μ*g/ml hydrocortisone, 100 ng/ml cholera toxin, 20 ng/ml EGF (all supplied by Sigma, France), and 1% penicillin/streptomycin (Life Technologies). This medium will then be referred as ‘MCF10A cells medium'. The cell line was tested for mycoplasma contamination before conducting the experiments.

The chronically exposed cellular model was obtained by incubating MCF10A cells with 10^−10^ M bisphenol A or 10^−10^ M benzo(a)pyrene for 60 days in phenol red-free DMEM/F-12 medium supplemented with 5% steroid-depleted, dextran-coated and charcoal-treated horse serum, 10 *μ*g/ml insulin, 0.5 *μ*g/ml hydrocortisone, 100 ng/ml cholera toxin, 20 ng/ml EGF (all supplied by Sigma), and 1% penicillin/streptomycin (Life Technologies). Media and treatments were changed every 2 days. This led to the establishment of three distinct MCF10A cell sublines (MCF10A-CT, MCF10A-BPA, MCF10A-B(a)P).

For the BMP treatment, serum contained in the MCF10A cells medium was reduced to 2%, and BMP2 or BMP4 were used at a concentration of 15 ng/ml for 4 days. For mammosphere assays, single cells were seeded onto 96-well ultra-low attachment plates (BD Corning, France) at limiting dilutions (150 cells/96-plate well) in medium as described previously.^9^ For the E-CFC assay, cells were seeded in MCF10A cells 2% serum medium at a limiting dilution (200 cells per well/12-well plate) as described previously by our team.^22^ Colonies were classified and counted following established size and morphological criteria.^22, 42^ For three dimensional TLDU assays, 500 cells were seeded in growth factor-reduced Matrigel (BD Corning), and assays were carried out in MCF10A cells medium.^21^ Structures were then washed in PBS 1 ×, fixed in 1% formaldehyde for 2 h, and sent to the ANIPATH platform (Lyon, France) for embedding, sectioning and H&E staining.

Primary cells were obtained from human adult breast reduction mammoplasties (informed consent was obtained from the patients) as described previously.^21^ To perform sphere-forming assays, total epithelial cell suspensions were grown in sphere culture medium using ultra-low attachment plates (BD Corning) for 7 days, and BMP2 or BMP4 were included at a concentration of 15 ng/ml. Resulting spheres were then dissociated into single cell suspension for cell proliferation and viability quantifications.

### Immunohistochemistry

IHC staining of paraffin sections of normal human breast tissue and primary breast tumors from Centre Léon Bérard was carried out using standard methods and the following antibody was used for the labeling: BMPR1B (ab78417), as described previously.^9^

### Flow cytometry

Cells were suspended in PBS 1 × and incubated for 30 min to 1 h with the relevant antibody following the manufacturer's instructions: FITC-conjugated anti-BMPR1a (R&D systems, Mineapolis, MN, USA) (or isotype FITC-conjugated IgG1), PE-conjugated anti-BMPR1b (R&D systems) (or isotype PE-conjugated IgG2B). Flow cytometry was performed using a FACSCalibur cell analyzer (BD Biosciences, Franklin lakes, NJ, USA).

### Western blot analysis

Cells were lysed in RIPA buffer (50 mM Tris, pH 7.4, 150 mM, NaCl, 5 mM EDTA, pH 8, 30 mM NaF, 1 mM Na_3_VO_4_, 40 mM *β*-glycerophosphate, protease inhibitors cocktail, Roche, France). Whole-cell extracts were fractionated by SDS-PAGE and transferred onto a polyvinylidene difluoride membrane using a transfer apparatus according to the manufacturer's protocols (Bio-Rad Trans Blot Turbo, Hercules, CA, USA). After incubation with 5% nonfat milk in TBST (10 mM Tris, pH 8, 150 mM NaCl, 0.5% Tween 20) for 30 min, the membrane was washed once with TBST and incubated with antibodies at 4 °C for 12 h, as detailed in the table below. Membranes were washed three times for 10 min and incubated with horseradish peroxidase-conjugated anti-mouse or anti-rabbit antibodies (Jackson Research, West Grove, PA, USA) at a dilution of 1:25 000 for 45 min. Blots were washed with TBST three times and developed with the ECL system (Roche Lumi-Light Plus) according to the manufacturer's guidelines.


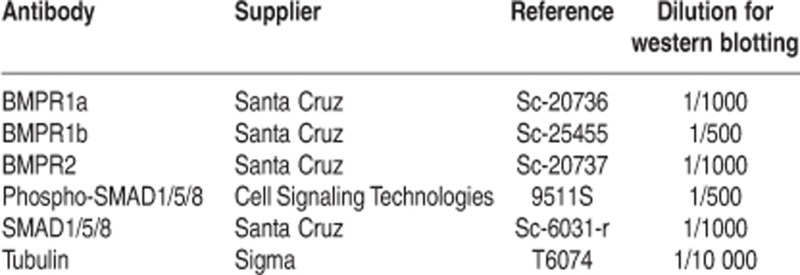


### Gene expression analysis

RNA was extracted using the RNeasy Plus Mini Kits (Qiagen, Valencia, CA, USA) containing a gDNA eliminator column. RNA concentration was measured using a Nanodrop ND-1000 spectrophotometer (Thermo Scientific, France). Reverse transcription was conducted using Superscript II (Invitrogen, France) according to the manufacturer's instructions. The cDNA was stored at −80 °C until further use. qPCR was performed using sequence-specific primers on a LightCycler 480 II system (Roche Applied Science, Indianapolis, IN, USA) with SyBR Green I technology (QuantiFAST SyBR kit from Qiagen) and LightCycler 480 Multiwell Plates 96 (Roche Applied Science). CPB and ACTB1 were selected by geNorm analysis as reference genes.


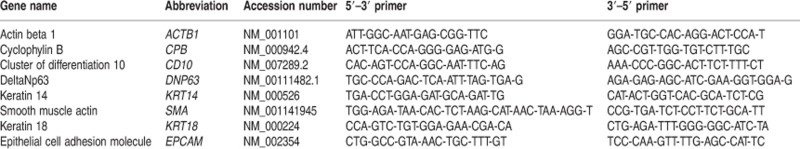


### Statistical analyses

Treated cells were compared with untreated cells using the paired Student's *t*-test, when data were normally distributed, or the Wilcoxon signed-rank test when data were not normally distributed, using *α*=0.05. For all of the experiments, significance was set at a *P*-value of *P<0.05.

## Abbreviations

BMP: bone morphogenetic protein
SC: stem cell
BPA: bisphenol A
B(a)P: benzo(a)pyrene
SMAD: small mothers against decapentaplegic
TGF*β*: transforming growth factor beta
BMPR2: bone morphogenetic protein receptor type 2
E-CFC: epithelial-colony forming cell
TDLU: terminal duct lobular unit
CD: cluster of differentiation
DNP63: deltaNp63
KRT: keratin
SMA: smooth muscle actin
EPCAM: epithelial cell adhesion molecule

## References

[bib1] Romero A, Prat A, Garcia-Saenz JA, Del Prado N, Pelayo A, Furió V et al. Assignment of tumor subtype by genomic testing and pathologic-based approximations: implications on patient's management and therapy selection. Clin Transl Oncol 2014; 16: 386–394. 23907291 10.1007/s12094-013-1088-z

[bib2] Brody JG, Rudel RA, Michels KB, Moysich KB, Bernstein L, Attfield KR et al. Environmental pollutants, diet, physical activity, body size, and breast cancer: where do we stand in research to identify opportunities for prevention? Cancer 2007; 109: 2627–2634. 17503444 10.1002/cncr.22656

[bib3] Goodson WH 3rd, Lowe L, Carpenter DO, Gilbertson M, Manaf Ali A, Lopez de Cerain Salsamendi A et al. Assessing the carcinogenic potential of low-dose exposures to chemical mixtures in the environment: the challenge ahead. Carcinogenesis 2015; 36: S254–S296. 26106142 10.1093/carcin/bgv039 PMC4480130

[bib4] Routledge EJ, White R, Parker MG, Sumpter JP. Differential effects of xenoestrogens on coactivator recruitment by estrogen receptor (ER) alpha and ERbeta. J Biol Chem 2000; 275: 35986–35993. 10964929 10.1074/jbc.M006777200

[bib5] Kasala ER, Bodduluru LN, Barua CC, Sriram CS, Gogoi R. Benzo(a)pyrene induced lung cancer: role of dietary phytochemicals in chemoprevention. Pharmacol Rep 2015; 67: 996–1009. 26398396 10.1016/j.pharep.2015.03.004

[bib6] Fernandez SV, Russo J. Estrogen and xenoestrogens in breast cancer. Toxicol Pathol 2010; 38: 110–122. 19933552 10.1177/0192623309354108 PMC2907875

[bib7] LaPensee EW, LaPensee CR, Fox S, Schwemberger S, Afton S, Ben-Jonathan N. et al. Bisphenol A and estradiol are equipotent in antagonizing cisplatin-induced cytotoxicity in breast cancer cells. Cancer Lett 2010; 290: 167–173. 19796866 10.1016/j.canlet.2009.09.005 PMC2853868

[bib8] Shim Y, Song JM. Spectral overlap-free quantum dot-based determination of benzo[a]pyrene-induced cancer stem cells by concurrent monitoring of CD44, CD24 and aldehyde dehydrogenase 1. Chem Commun 2015; 51: 2118–2121. 10.1039/c4cc08953g 25536409

[bib9] Chapellier M, Bachelard-Cascales E, Schmidt X, Clément F, Treilleux I, Delay E et al. Disequilibrium of BMP2 levels in the breast stem cell niche launches epithelial transformation by overamplifying BMPR1B cell response. Stem Cell Rep 2015; 4: 239–254. 10.1016/j.stemcr.2014.12.007 PMC4325271 25601208

[bib10] Casey SC, Vaccari M, Al-Mulla F, Al-Temaimi R, Amedei A, Barcellos-Hoff MH et al. The effect of environmental chemicals on the tumor microenvironment. Carcinogenesis 2015; 36: S160–S183. 26106136 10.1093/carcin/bgv035 PMC4565612

[bib11] Marcucci F, Rumio C, Lefoulon F. Anti-cancer stem-like cell compounds in clinical development—an overview and critical appraisal. Front Oncol 2016; 6: 115. 27242955 10.3389/fonc.2016.00115 PMC4861739

[bib12] Yadin D, Knaus P, Mueller TD. Structural insights into BMP receptors: specificity, activation and inhibition. Cytokine Growth Factor Rev 2016; 27: 13–34. 26690041 10.1016/j.cytogfr.2015.11.005

[bib13] Kawabata M, Inoue H, Hanyu A, Imamura T, Miyazono K. Smad proteins exist as monomers *in vivo* and undergo homo- and hetero- oligomerization upon activation by serine/threonine kinase receptors. Embo J 1998; 17: 4056–4065. 9670020 10.1093/emboj/17.14.4056 PMC1170738

[bib14] Massague J, Wotton D. Transcriptional control by the TGF-beta/Smad signaling system. Embo J 2000; 19: 1745–1754. 10775259 10.1093/emboj/19.8.1745 PMC302010

[bib15] Alarmo EL, Kallioniemi A. Bone morphogenetic proteins in breast cancer: dual role in tumourigenesis? Endocr Relat Cancer 2010; 17: R123–R139. 20335308 10.1677/ERC-09-0273

[bib16] Ye L, Bokobza SM, Jiang WG. Bone morphogenetic proteins in development and progression of breast cancer and therapeutic potential (review). Int J Mol Med 2009; 24: 591–597. 19787192 10.3892/ijmm_00000269

[bib17] Ye L, Mason MD, Jiang WG. Bone morphogenetic protein and bone metastasis, implication and therapeutic potential. Front Biosci 2011; 16: 865–897. 10.2741/3725 21196208

[bib18] Tan EJ, Olsson A-K, Moustakas A. Reprogramming during epithelial to mesenchymal transition under the control of TGFβ. Cell Adh Migr 2015; 9: 233–246. 25482613 10.4161/19336918.2014.983794 PMC4594534

[bib19] Mohrin M, Bourke E, Alexander D, Warr MR, Barry-Holson K, Le Beau MM et al. Hematopoietic stem cell quiescence promotes error-prone DNA repair and mutagenesis. Cell Stem Cell 2010; 7: 174–185. 20619762 10.1016/j.stem.2010.06.014 PMC2924905

[bib20] Neve RM, Chin K, Fridlyand J, Yeh J, Baehner FL, Fevr T et al. A collection of breast cancer cell lines for the study of functionally distinct cancer subtypes. Cancer Cell 2006; 10: 515–527. 17157791 10.1016/j.ccr.2006.10.008 PMC2730521

[bib21] Bachelard-Cascales E, Chapellier M, Delay E, Pochon G, Voeltzel T, Puisieux A et al. The CD10 enzyme is a key player to identify and regulate human mammary stem cells. Stem Cells 2010; 28: 1081–1088. 20506111 10.1002/stem.435

[bib22] Clément F, Zhu HH, Gao WQ, Delay E, Maguer-Satta V. Quantifying epithelial early common progenitors from long-term primary or cell line sphere culture. Curr Protoc Stem Cell Biol 2015; 35: E71–E78. 10.1002/9780470151808.sc01e07s35 26544537

[bib23] Chapellier M, Maguer-Satta V. BMP2, a key to uncover luminal breast cancer origin linked to pollutant effects on epithelial stem cells niche. Mol Cell Oncol 2016; 3: e1026527. 27314065 10.1080/23723556.2015.1026527 PMC4909443

[bib24] Jeanpierre S, Nicolini FE, Kaniewski B, Dumontet C, Rimokh R, Puisieux A et al. BMP4 regulation of human megakaryocytic differentiation is involved in thrombopoietin signaling. Blood 2008; 112: 3154–3163. 18664625 10.1182/blood-2008-03-145326

[bib25] Laperrousaz B, Jeanpierre S, Sagorny K, Voeltzel T, Ramas S, Kaniewski B et al. Primitive CML cell expansion relies on abnormal levels of BMPs provided by the niche and on BMPRIb overexpression. Blood 2013; 122: 3767–3777. 24100446 10.1182/blood-2013-05-501460

[bib26] Sengupta S, Obiorah I, Maximov PY, Curpan R, Jordan VC. Molecular mechanism of action of bisphenol and bisphenol A mediated by oestrogen receptor alpha in growth and apoptosis of breast cancer cells. Br J Pharmacol 2013; 169: 167–178. 23373633 10.1111/bph.12122 PMC3632247

[bib27] Mlynarcikova A, Macho L, Fickova M. Bisphenol A alone or in combination with estradiol modulates cell cycle- and apoptosis-related proteins and genes in MCF7 cells. Endocr Regul 2013; 47: 189–199. 24156707 10.4149/endo_2013_04_189

[bib28] Zhang W, Fang Y, Shi X, Zhang M, Wang X, Tan Y. Effect of bisphenol A on the EGFR-STAT3 pathway in MCF-7 breast cancer cells. Mol Med Rep 2012; 5: 41–47. 21909620 10.3892/mmr.2011.583

[bib29] Fourel L, Valat A, Faurobert E, Guillot R, Bourrin-Reynard I, Ren K et al. beta3 integrin-mediated spreading induced by matrix-bound BMP-2 controls Smad signaling in a stiffness-independent manner. J Cell Biol 2016; 212: 693–706. 26953352 10.1083/jcb.201508018 PMC4792076

[bib30] Soto AM, Brisken C, Schaeberle C, Sonnenschein C. Does cancer start in the womb? Altered mammary gland development and predisposition to breast cancer due to *in utero* exposure to endocrine disruptors. J Mammary Gland Biol Neoplasia 2013; 18: 199–208. 23702822 10.1007/s10911-013-9293-5 PMC3933259

[bib31] Ketolainen JM, Alarmo EL, Tuominen VJ, Kallioniemi A. Parallel inhibition of cell growth and induction of cell migration and invasion in breast cancer cells by bone morphogenetic protein 4. Breast Cancer Res Treat 2010; 124: 377–386. 20182795 10.1007/s10549-010-0808-0

[bib32] Alarmo EL, Huhtala H, Korhonen T, Pylkkänen L, Holli K, Kuukasjärvi T et al. Bone morphogenetic protein 4 expression in multiple normal and tumor tissues reveals its importance beyond development. Mod Pathol 2013; 26: 10–21. 22899288 10.1038/modpathol.2012.128

[bib33] Ampuja M, Jokimaki R, Juuti-Uusitalo K, Rodriguez-Martinez A, Alarmo EL, Kallioniemi A. BMP4 inhibits the proliferation of breast cancer cells and induces an MMP-dependent migratory phenotype in MDA-MB-231 cells in 3D environment. BMC Cancer 2013; 13: 429. 24053318 10.1186/1471-2407-13-429 PMC3848934

[bib34] Guo D, Huang J, Gong J. Bone morphogenetic protein 4 (BMP4) is required for migration and invasion of breast cancer. Mol Cell Biochem 2012; 363: 179–190. 22167620 10.1007/s11010-011-1170-1

[bib35] Cao Y, Slaney CY, Bidwell BN, Parker BS, Johnstone CN, Rautela J et al. BMP4 inhibits breast cancer metastasis by blocking myeloid-derived suppressor cell activity. Cancer Res 2014; 74: 5091–5102. 25224959 10.1158/0008-5472.CAN-13-3171

[bib36] Huang WC, Chen YJ, Li LY, Wei YL, Hsu SC, Tsai SL et al. Nuclear translocation of epidermal growth factor receptor by Akt-dependent phosphorylation enhances breast cancer-resistant protein expression in gefitinib-resistant cells. J Biol Chem 2011; 286: 20558–20568. 21487020 10.1074/jbc.M111.240796 PMC3121497

[bib37] Dayde D, Guerard M, Perron P, Hatat AS, Barrial C, Eymin B et al. Nuclear trafficking of EGFR by Vps34 represses Arf expression to promote lung tumor cell survival. Oncogene 2016; 30: 3986–3994. 10.1038/onc.2015.480 26686095

[bib38] Fremin C, Bessard A, Ezan F, Gailhouste L, Régeard M, Le Seyec J et al. Multiple division cycles and long-term survival of hepatocytes are distinctly regulated by extracellular signal-regulated kinases ERK1 and ERK2. Hepatology 2009; 49: 930–939. 19177593 10.1002/hep.22730

[bib39] Chen JY, Lin JR, Cimprich KA, Meyer T. A two-dimensional ERK-AKT signaling code for an NGF-triggered cell-fate decision. Mol Cell 2012; 45: 196–209. 22206868 10.1016/j.molcel.2011.11.023 PMC3897208

[bib40] Zi Z, Feng Z, Chapnick DA, Dahl M, Deng D, Klipp E et al. Quantitative analysis of transient and sustained transforming growth factor-beta signaling dynamics. Mol Syst Biol 2011; 7: 492. 21613981 10.1038/msb.2011.22 PMC3130555

[bib41] Pluchino LA, Wang HC. Chronic exposure to combined carcinogens enhances breast cell carcinogenesis with mesenchymal and stem-like cell properties. PLoS One 2014; 9: e108698. 25372613 10.1371/journal.pone.0108698 PMC4220909

[bib42] Stingl J, Raouf A, Emerman JT, Eaves CJ. Epithelial progenitors in the normal human mammary gland. J Mammary Gland Biol Neoplasia 2005; 10: 49–59. 15886886 10.1007/s10911-005-2540-7

