# Supplementary figures and images for: Long-term exposure to bisphenol A or benzo(a)pyrene alters the fate of human mammary epithelial stem cells in response to BMP2 and BMP4, by pre-activating BMP signaling
Source: Cell Death Differ. 2016 Oct 14;24(1):155–66. doi: 10.1038/cdd.2016.107 (PMC5260492; doi:10.1038/cdd.2016.107)

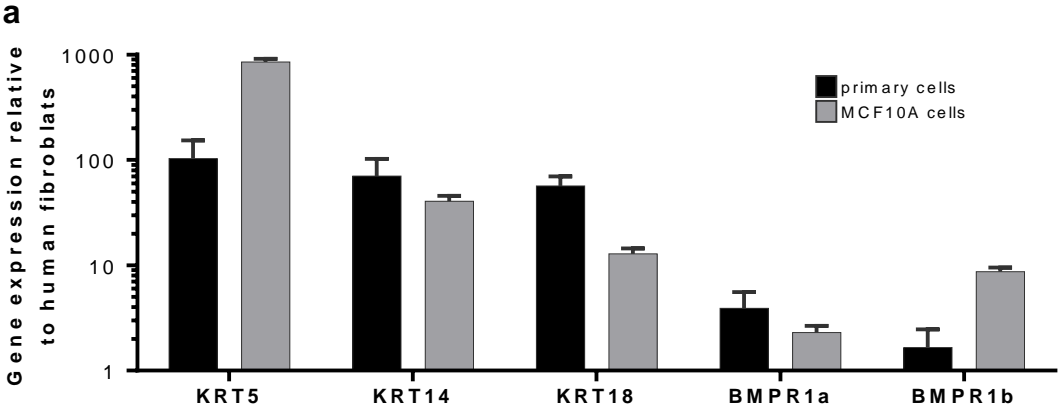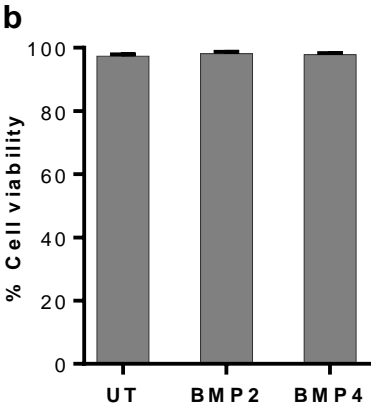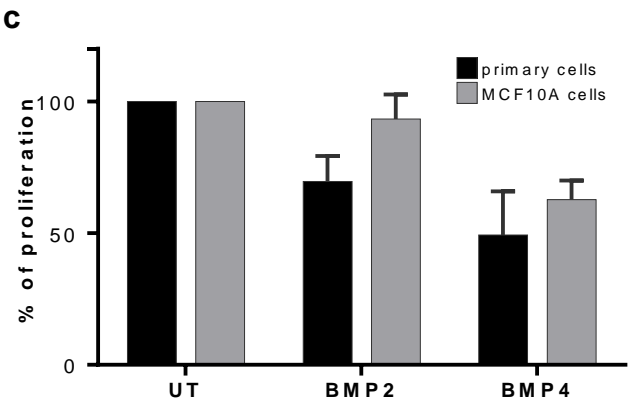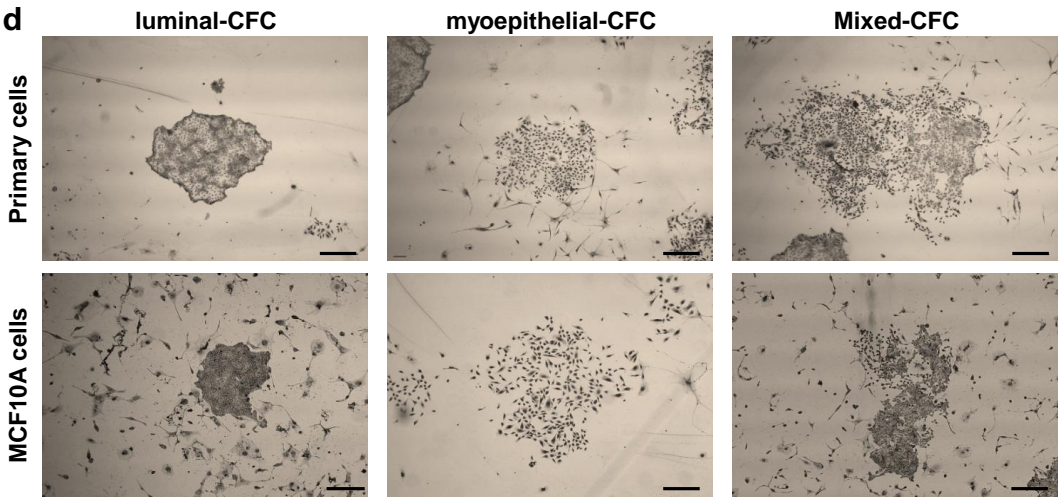

Supplement: Supplementary Figure 1 [file cdd2016107x1.pdf]
